# UV Filters, Ingredients with a Recognized Anti-Inflammatory Effect

**DOI:** 10.1371/journal.pone.0046187

**Published:** 2012-12-20

**Authors:** Céline Couteau, Catherine Chauvet, Eva Paparis, Laurence Coiffard

**Affiliations:** 1 Université de Nantes, Nantes Atlantique Universités, MMS, EA 2160, FR CNRS 3473 - Faculty of Pharmacy, Nantes, France; 2 Université de Nantes, Nantes Atlantique Universités, Pharmacochemistry Department, Faculty of Pharmacy, Nantes University, Nantes Atlantique Universities, IICiMed UPRES EA 1155, Nantes, France; CNRS-University of Toulouse, France

## Abstract

**Background:**

To explain observed differences during SPF determination using either an *in vivo* or *in vitro* method, we hypothesized on the presence of ingredients having anti-inflammatory properties.

**Methodology/Principal Findings:**

To research our hypothesis, we studied the 21 UV filters both available on the market and authorized by European regulations and subjected these filters to the phorbol-myristate-acetate test using mice. We then catalogued the 13 filters demonstrating a significant anti-inflammatory effect with edema inhibition percentages of more than 70%. The filters are: diethylhexyl butamido triazone (92%), benzophenone-5 and titanium dioxide (90%), benzophenone-3 (83%), octocrylène and isoamyl p-methoxycinnamate (82%), PEG-25 PABA and homosalate (80%), octyl triazone and phenylbenzimidazole sulfonic acid (78%), octyl dimethyl PABA (75%), bis-ethylhexyloxyphenol methoxyphenyl triazine and diethylamino hydroxybenzoyl hexylbenzoate (70%). These filters were tested at various concentrations, including their maximum authorized dose. We detected a dose-response relationship.

**Conclusions/Significance:**

The anti-inflammatory effect of a sunscreen ingredient may affect the in vivo SPF value.

## Introduction

The effectiveness of sunscreen products is quantifiable using two indicators, the SPF (*Sun Protection Factor*) and the UVA-PF (*UVA-Protection Factor*). For many years, these two indicators were determined *in vivo* using volunteers [1], [2]. For ethical reasons, *in vitro* methods have been more recently proposed in order to spare humans from excessive sun exposure [3]–[6]. While these two methods are perfectly correlated in a great number of situations, it has been demonstrated that with respect to SPF determination for many products, the *in vivo* values are higher than those obtained for *in vitro* method. This difference can be explained by the use of certain ingredients having anti-inflammatory properties to formulate these products, such as α-bisabolol and 18 β-glycyrrhetinic acid [7]–[10]. Thus, the goal of our research was to determine the intrinsic anti-inflammatory properties of the UV filters currently authorized for use in Europe.

## Materials and Methods

Paraffinum liquidum, Cetiol® HE, stearic acid, glycerin, parabens and triethanolamin (TEA) were purchased from Cooper (Melun, France). Xanthan gum (Rhodicare® T) was obtained from Rhodia (Paris, France). Polymethylmethacrylate (PMMA) plates were purchased from Europlast (Aubervilliers, France). The standard (reference) molecules Phorbol 12-Myristate 13-Acetate (PMA), niflumic acid, hydrocortisone 17-butyrate, diclofenac and ketoprofen were purchased from Sigma Aldrich (Saint Quentin Fallavier, France). The filters we tested are presented in Table 1. Note that we also studied zinc oxide (Tegosun® Z500, Goldschmidt, Montigny-le-Bretonneux), an ingredient that is not included in the list of Europe-authorized filters but traditionally used in sunscreen products. We tested thirteen commercial sunscreen products, with varying levels of protection (weak, medium and very high) (Table 2).

**Table 1:** Characteristics of UV-filters tested.

INCI Name	Trade name (Suppliers)
Homosalate	Neoheliopan HMS (Symrise, Puteaux, France)
Oxybenzone	Neoheliopan BB (Symrise, Puteaux, France)
Phenylbenzimidazole sulfonic acid	Eusolex 232 (Merck, Fontenay sous Bois, France)
Butylmethoxydibenzoylmethane	Eusolex 9020 (Merck, Fontenay sous Bois, France)
Octocrylene	Parsol 340 (Roche vitamines, Neuilly/Seine, France)
Ethyl hexyl methoxycinnamate	Escalol 557 (International speciality products, Köln, Germany)
PEG-25 PABA	Uvinul P25 (Laserson, Etampes, France)
Isoamyl p-methoxycinnamate	Neoheliopan E1000 (Symrise, Puteaux, France)
Octyl triazone	Uvinul T150 (Laserson, Etampes, France)
Diethylhexylbutamidotriazone	Uvasorb HEB (3 V Sigma, Bergamo, Italy)
4-methylbenzylidene camphor	Neoheliopan MBC (Symrise, Puteaux, France)
Ethylhexylsalicylate	Eusolex OS (Merck, Fontenay/Bois, France)
Octyldimethyl PABA	Escalol 507 (International speciality products, Köln, Germany)
Benzophenone-5	Uvinul MS 40 (Laserson, Etampes, France)
Methylene bis-benzotriazolyl tetramethylbutylphenol	Tinosorb M (Ciba, Saint-Fons, France)
Disodium phenyl dibenzimidazole tetrasulfonate	Neoheliopan AP (Symrise, Puteaux, France)
Bis-Ethylhexyloxyphenol methoxyphenyl triazine	Tinosorb S (Ciba, Saint-Fons, France)
Polysilicone 15	Parsol SLX (Roche vitamines, Neuilly/Seine, France)
Diethylamino hydroxybenzoyl hexyl benzoate	Uvinul A+ (Laserson, Etampes, France)
Titanium dioxide, Butylene glycol dicaprylate/dicaprate, Silica, Polyglyceryl-2 dipolyhydroxystearate	Eusolex T-Oleo (Merck, Fontenay/Bois, France)
Zinc oxide	Tegosun Z 500 (Degussa, Essen, Germany)

**Table 2:** Sunscreen products tested.

Trade name/labelled SPF	UV-filters
Nivea Sun 50+	Octocrylene, Butylmethoxydibenzoylmethane, Bis-ethylhexyloxyphenol methoxyphenyl triazine, Homosalate, Titanium dioxide, Sodium phenylbenzimidazole sulfonate, Diethylhexyl butamido triazone, Ethylhexylmethoxycinnamate
Vichy Capital soleil® lotion **50+**	Octocrylene, Ethylhexylsalicylate, Butylmethoxydibenzoylmethane, Titanium dioxide, Ethylhexyltriazone, Terephyalydene dicamphor sulfonic acid,
Bioderma Photoderm bronz spray **15**	Octocrylene, Butylmethoxydibenzoylmethane, Methylene bis-benzotriazolyl tetramethylbutylphenol
La Roche Posay spray lotion **20**	Octocrylene, Butylmethoxydibenzoylmethane, Ethylhexyltriazone, Drometrisole trisiloxane, Terephtalydene dicamphor sulfonic acid, Titanium dioxide
Roc soleil protexion® lotion spray **30**	Ethylhexylsalicylate, Octocrylene, Butylmethoxydibenzoylmethane, Bis-Ethylhexyloxyphenol methoxyphenyl triazine, Diethylamino hydroxybenzoyl hexyl benzoate, Methylene bis-benzotriazolyl tetramethylbutylphenol
Avene spray sensitive skin **50+**	Methylene bis-benzotriazolyl tetramethylbutylphenol, Titanium dioxide, Titanium dioxide, Butylmethoxydibenzoylmethane, Bis-Ethylhexyloxyphenol methoxyphenyl triazine
Lancaster Oil-free milky spray **6**	Bis-ethylhexyloxyphenol methoxyphenyl triazine, Butylmethoxydibenzoylmethane, Octocrylene, Ethylhexylmethoxycinnamate
Soleil Biafine® sun lotion spray **30**	Octocrylene, Methylene bis-benzotriazolyl tetramethylbutylphenol, Butylmethoxydibenzoylmethane, Diethylamino hydroxybenzoyl hexyl benzoate, Bis-Ethylhexyloxyphenol methoxyphenyl triazine
Polysianes milky spray **30**	Ethylhexylmethoxycinnamate, Octocrylene, Methylene bis-benzotriazolyl tetramethylbutylphenol, Bis-Ethylhexyloxyphenol methoxyphenyl triazine
Sun lotion kids Carrefour **50+**	Octocrylene, Titanium dioxide, Butylmethoxydibenzoylmethane, Bis-Ethylhexyloxyphenol methoxyphenyl triazine, Phenylbenzimidazole sulfonic acid, Diethylhexyl butamidotriazone
Vea scudo SPF **5**	Titanium dioxide, Zinc oxide
Solei^SP^ Boots SPF **15**	Octocrylene, Butyl methoxydibenzoylmethane, Ethylhexyl salicylate, Diethylhexylbutamidotriazone, Bis-ethylhexyloxyphenol methoxyphenyl triazine, Titanium oxide
Hello Kitty SPF **30**	Homosalate, Ethylhexylsalicylate, Benzophenone-3, Butyl methoxydibenzoylmethane

Swiss mice, male, weighing 14–16 grams, were purchased from Janvier (St Berthevin, France). The mice were bred in the animal facility of the department of Pharmacology. The mice were caged individually and were fed standard laboratory chow and water *ad libitum* under standard conditions of temperature and light.

An O/W emulsion placebo was prepared in the laboratory as previously described [11]. The different filters and standard (reference) molecules were incorporated into the formulation in order to study their anti-inflammatory properties. Concerning the incorporation percentages, for the anti-inflammatory reference molecules, we followed the dose commonly found in medicinal preparations that use them; for the filters, we decided to use the maximum authorized dose with respect to current European regulations. However, for the filters that demonstrated a noteworthy anti-inflammatory effect by inhibiting the edema more than 70%, the dose-response relationship was also studied via other concentrations such as 0.50, 1.25, 2.50 and 5.00%. For titanium dioxide, which is authorized up to 25.0%, we also tested formulas containing 2.5, 5.0, 10.0 and 15.0% of this ingredient. Hydrophilic phase and oil-phase were heated separately to 78 to 82°C, until the contents of each part were solubilized. Then, the oily preparation was added slowly to the hydrophilic preparation while stirring (Yellow line OST basic mixer, IKA, Staufen, Germany). It was necessary to continue stirring until the emulsion formed was cooled to room temperature (20°C).

The SPF of the commercial sunscreen products was determined using an *in vitro* method according to a previously described protocol (Couteau *et al*, 2007a). 30 mg of product was weighed and then spread across the entire surface of PMMA plates (25 cm^2^) using a finger cot. 15 mg remained on the finger cot. Three plates were prepared for each product to be tested and 9 measures were performed on each plate. Transmission measurements between 290 and 400 nm were taken using a spectrophotometer equipped with a xenon arc lamp and an integrating sphere (UV Transmittance Analyzer UV1000S, Labsphere, North Sutton, US). The standard used was the 8% homosalate standard mandated by the US Food and Drug Administration Sunscreen Monograph. The calculations for either term used the same relationship:
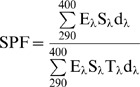
where E_λ_ is the CIE erythemal spectral effectiveness, S_λ_ is the solar spectral irradiance and T_λ_ is the spectral transmittance of the sample [3].

We determined the anti-inflammatory effects of the emulsions formulated in the laboratory and the commercial sunscreen products using a Phorbol 12-Myristate 13-Acetate (PMA) test. The mouse ear edema was provoked according to the method described by Carlson et al. with some modifications [12]–[14]. First, the thickness of the mouse ears was measured using a model micrometer gauge (Oditest®, Kroeplin, Schlüchtern, Germany). 10 µL of sunscreen product or preparation with standard (reference) molecule or filter was applied using a ripette genix electro dispenser (Fisher scientific, Illkirch, France), to the mice's right ears, twice at 5 minute intervals. 10 µL of placebo emulsion was applied, following the same protocol, to the mice's left ears. Thirty minutes later, 10 µL of a hydro-alcoholic solution of Phorbol-12-Myristate-13-Acetate (250 µg.mL^−1^) was then applied to each ear, in order to provoke an edema. After three and a half hours, the Oditest® was performed again to determine the thickness of the ears. Ear edema, calculated by subtracting the thickness of the left ear (vehicle) from the thickness of the right ear (PMA), was expressed as an increase in ear thickness. We determined the percentage that the inflammatory reaction was inhibited for each mouse by comparing the ear edema in treated and untreated animals. Five mice were used for each product tested.

## Results and Discussion

The SPF values (*in vitro* determination) of the commercially-available sunscreen products are presented in Table 3. Six products presented an *in vitro* SPF value inferior to the labeled value.

**Table 3:** SPF of sunscreen products using in vitro method.

Trade name/labelled SPF	SPF (Mean ± SD)
Nivea Sun **50+**	76±8
Vichy Capital soleil® lotion **50+**	44±4
Bioderma Photoderm bronz spray **15**	17±2
La Roche Posay spray lotion **20**	19±2
Roc Soleil protexion® lotion spray **30**	18±2
Avene spray **50+**	41±4
Lancaster **6**	11±1
Soleil Biafine® lotion spray solaire **30**	24±2
Polysianes milky spray **30**	48±4
Sun lotion kids Carrefour **50+**	57±3
Vea scudo SPF **5**	3±0
Solei^SP^ Boots SPF **15**	15±1
Hello Kitty SPF **30**	17±1

The anti-inflammatory effect of the 4 standard molecules and the 21 UV filters (all substances applied topically to the mice) was studied. Note that the large majority of the filters demonstrated a significant anti-inflammatory effect (Tables 4 and 5). Two filters stood out from the rest: diethylhexyl butamido triazone and benzophenone-5. These two demonstrated the greatest anti-inflammatory properties, showing an effectiveness similar to ketoprofen. Titanium dioxide also revealed itself to be quite anti-inflammatory. However, it should be remembered that we tested this substance at its highest incorporation dose—25%, a level that is never attained in practice because of the problematic consistency of a product formulated with this percentage.

**Table 4:** Anti-inflammatory effect of references.

References	(% w/w)	Edema inhibition (%)Mean ± SD
Niflumic acid	2.5	99±2
Hydrocortisone 17-butyrate	0.1	99±2
Diclofenac	1.0	100±0
Ketoprofen	2.5	93±2

**Table 5:** Anti-inflammatory effect of UV-filters tested at the maximum authorized concentration.

Filters	(% w/w)	Edema inhibition (%) Mean ± SD
Homosalate	10	83±4
Benzophénone-3	10	90±4
Phenylbenzimidazole sulfonic acid	8	80±2
Butylmethoxydibenzoylmethane	5	58±5
Octocrylene	10	83±4
Ethyl hexyl methoxycinnamate	10	56±3
PEG-25 PABA	10	82±3
Isoamyl p-methoxycinnamate	10	83±4
Octyl triazone	5	81±6
Diethylhexylbutamidotriazone	10	95±4
4-methylbenzylidene camphor	4	65±2
Ethylhexylsalicylate	5	59±4
Octyldimethyl PABA	8	78±3
Benzophenone-5	5	92±3
Methylene bis-benzotriazolyl tetramethylbutylphenol	10	56±2
Disodium phenyl dibenzimidazole tetrasulfonate	10	69±7
Bis-Ethylhexyloxyphenol methoxyphenyl triazine	10	76±3
Polysilicone 15	10	69±2
Diethylamino hydroxybenzoyl hexyl benzoate	10	70±4
Titanium dioxide, Butylene glycol dicaprylate/dicaprate, Silica, Polyglyceryl-2 dipolyhydroxystearate	25	92±3
Zinc oxide	25	63±5

**Figure 1:**
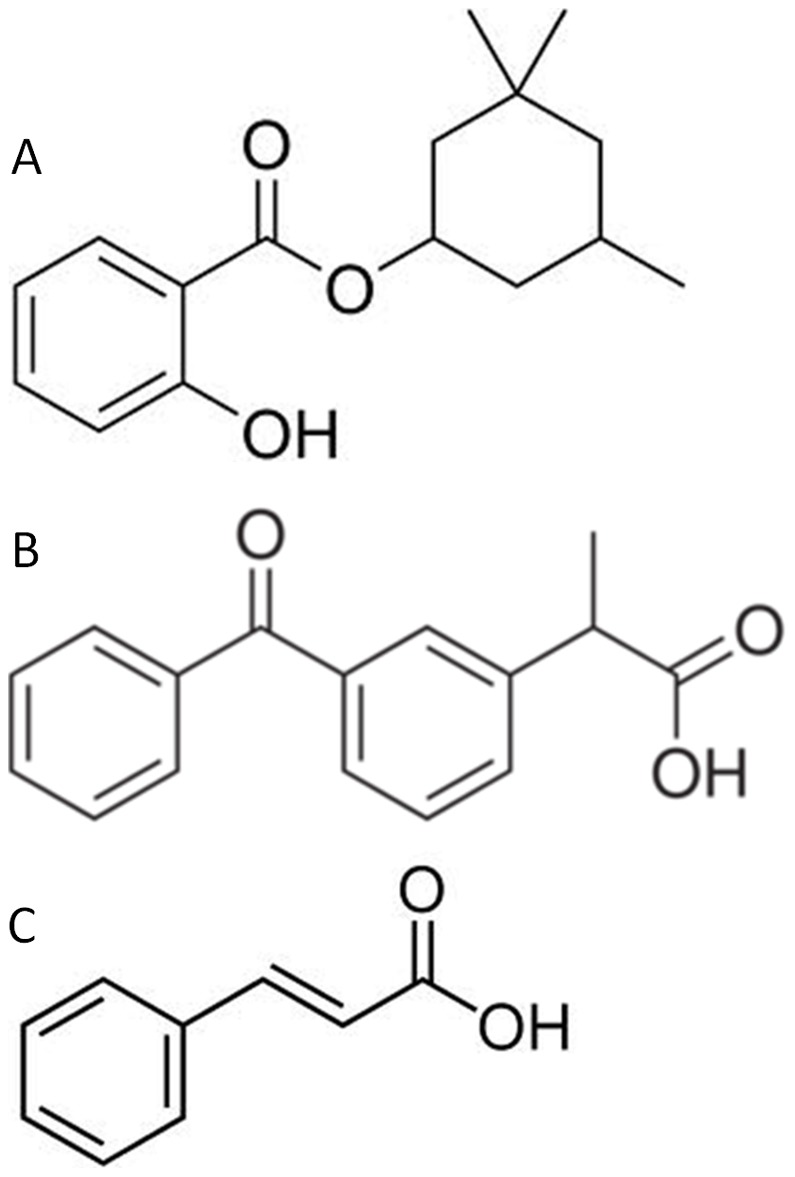
Molecular structures.

The activity of homosalate (Fig. 1) (Table 5) is not surprising as this filter belongs to the salicylate family, which are well-known non-steroidal anti-inflammatory substances (NSAIs) and some of which occur naturally [15]. The same is true for the benzophenones. Note that ketoprofen is a derivative of benzophenone (Fig. 1) and that numerous synthetic benzophenone substitutes have shown anti-inflammatory properties [16]–[19]. A structure-activity relationship of benzophenones as a novel class of MAP kinase inhibitors with high anti-inflammatory properties has been reported [20].

The anti-inflammatory effect of cinnamic acid derivatives (Fig. 1) such as caffeic acid is well known [21]–[23]. This would explain the results obtained with octyl methoxycinnamate and isoamyl p-methoxycinnamate. Furthermore, it would appear that benzimidazole type molecules also have an anti-inflammatory effect [24], [25]. Certain triazines demonstrated an inhibitory effect of the MAP kinases [26].

**Figure 2:**
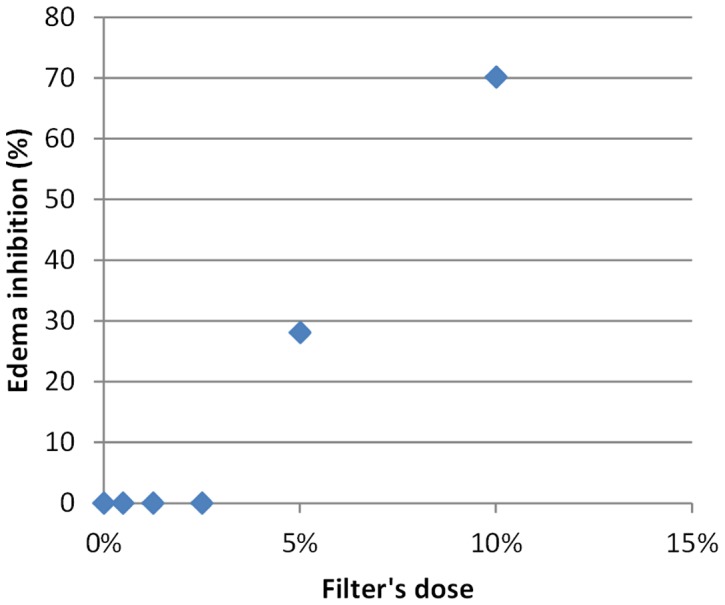
Dose Response Relationship for Diethylaminohydroxybenzoyl hexyl benzoate.

**Figure 3:**
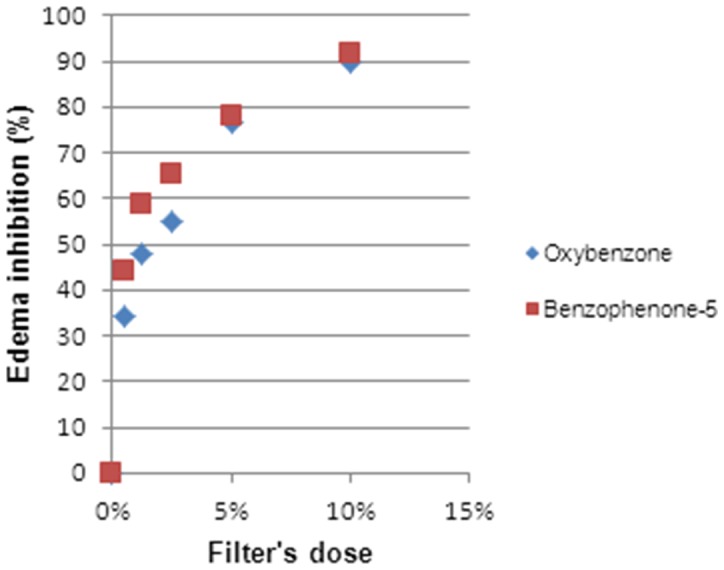
Dose Response Relationship for benzophenones.

**Figure 4:**
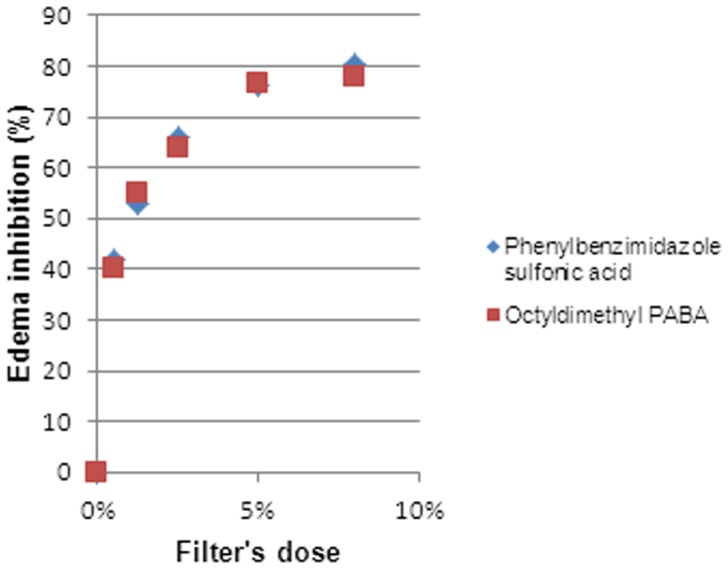
Dose Response Relationship for UV-filters limited to 8% (w/w).

**Figure 5:**
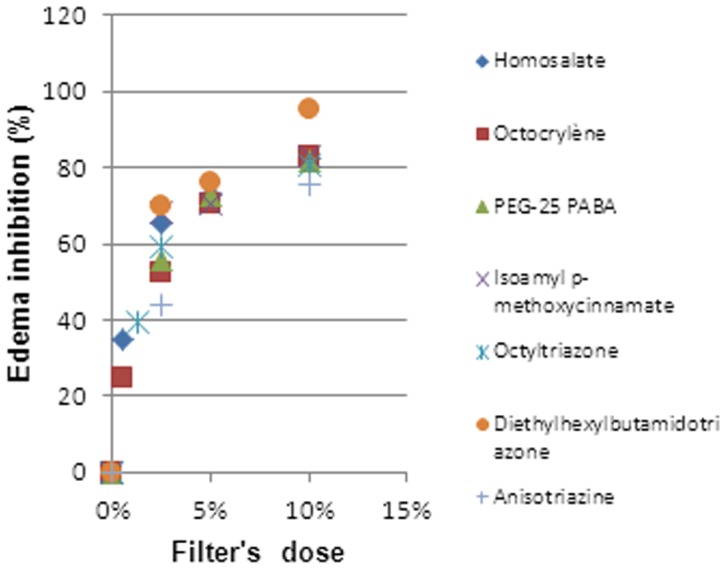
Dose Response Relationship for UV-filters limited to 10% (w/w).

**Figure 6:**
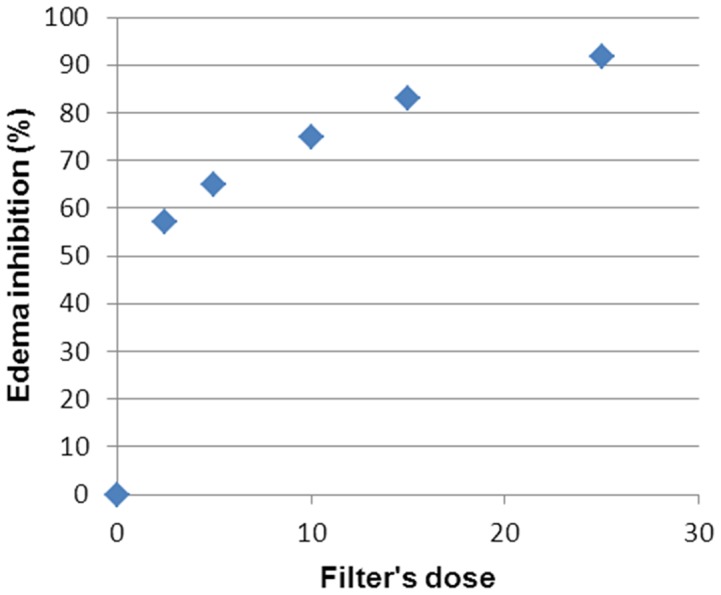
Dose Response Relationship for titanium dioxide.

We can clearly state that there exists a dose-response relationship (Fig. 2, 3, 4, 5 and 6). At a lower dose we observed a reduced anti-inflammatory effect. Only two filters (isoamyl p-methoxycinnamate and diethylhexyl butamido triazone), even at a low dose, inhibited the edema by about 70%.

We observed an anti-inflammatory effect with the commercial sunscreen products (Table 6). For half the products (six products on the thirteen studied), the effect was quite significant, with an edema inhibition upper to 70%.

**Table 6:** Anti-inflammatory effect of sunscreens.

Trade name	Edema inhibition (%) Mean ± SD
Nivea Sun 50+	76±3
Vichy capital soleil® lotion 50+	48±3
Bioderma Photoderm bronz spray 15	44±3
La Roche Posay spray lotion 20	85±6
Roc soleil protexion® lotion spray 30	80±1
Avene spray sensitive skin 50+	76±4
Lancaster oily-free milky spray 6	48±1
Soleil Biafine® sun lotion spray 30	48±4
Polysiane milky spray 30	71±5
Sun lotion kids Carrefour 50+	54±2
Vea scudo SPF 5	56±7
Solei^SP^ Boots SPF 15	74±2
Hello Kitty SPF 30	46±3

All of this has consequences in terms of determining the effectiveness of the final product. In Europe, it is difficult to determine whether a product is anti-inflammatory or not simply by reading the ingredients on the label because we do not know at what percentage these filters have been incorporated. The effect is not linked to the indicated protection factor, but to the specific filters and their concentrations. Certain sunscreen products, even with a medium protection level, like La Roche Posay® SPF 20 and Solei^SP^ Boots® SPF 15, for example, demonstrate a significant anti-inflammatory effect when compared to Vichy Capital soleil® Lait 50+. Besides, the anti-inflammatory effects can be modified when sunscreen is UV-exposed, as many UV-filters are known to be photo-unstable [11].

It is important to note that the *in vivo* method of SPF testing takes the anti-inflammatory effect of the products tested into consideration while the *in vitro* method does not. It remains to be shown how the biological response of the human UV-irradiated skin might vary with inclusion of specific anti-inflammatory ingredients in the sunscreens.
